# Precarious Projects: Conversions of (Biomedical) Knowledge in an East African City

**DOI:** 10.1080/01459740.2013.833918

**Published:** 2014-02-01

**Authors:** Ruth J. Prince

**Affiliations:** Centre of African Studies, University of Cambridge, Cambridge, United Kingdom, and Department of Anthropology, University of Oslo, Oslo, Norway

**Keywords:** documents, global health, identity, space, temporality

## Abstract

This article explores the orientations of lay people in Kenya to science—specifically to biomedical knowledge about HIV—and their struggles to convert this knowledge into meaningful futures. In Kenya, the global response to the HIV-AIDS epidemic has resulted in a highly stratified landscape of intervention. Globally-funded treatment programs and clinical trials, focusing on HIV, channel transnational resources, expertise, and knowledge into specific sites—HIV clinics, NGOs, and research stations—inscribing these spaces as ‘global’ while leaving others decidedly ‘local.’ Rolled out in the form of ‘projects,’ these interventions offer resources and opportunities for a limited time only. Based on ethnographic fieldwork in the city of Kisumu, this article follows the circulation of biomedical knowledge through such projects and its conversion in ways beyond those imagined by policy-makers, as it meets the aspirations of city-dwellers and enters into local livelihoods. Mediated by nongovernmental organizations through workshops and certificates, this knowledge is both fragmentary and ephemeral. I explore the temporal and spatial implications of such knowledge for those who seek to attach themselves to it and shape their identities and futures in relation to it.

And I remember when I got [my certificate]…. I never put it down I walked with it for almost like … four days, I just admired it and, ah, I could feel proud of myself because… because I had secured one thing that had taken me a long time. (Elias, HIV counselor, Kisumu, December 2008)

Elias is a young man in his early thirties living in the city of Kisumu in western Kenya. When I met him in November 2008, he had just been employed as an “HIV counselor” at a national nongovernmental organization (NGO) called Youth for Life, whose activities focused on HIV and reproductive health among Kenyan youth.^1^ He had been working for this organization and other NGOs as a largely unpaid volunteer for much of the previous five years. During this time the NGO sent him to various workshops that aimed to ‘disseminate’ knowledge about HIV and to train lay people in HIV education, ‘outreach’ work, counseling, and testing. Elias managed to gain several certificates relating to HIV work from his attendance at these workshops. He also signed up as a research subject for a large clinical trial investigating the effect of male circumcision on the risk of HIV infection, and he became a member of the NGO's youth group and got involved in their projects providing health education to the city's youth. He lived in a single room with a friend and gained some income from attending workshops, from the few hundred shillings a month he collected when attending the male circumcision clinic, from his activities as a ‘volunteer’ with the youth group, from the extra tuition he gave to primary school students, and from occasional positions he found within youth ‘outreach’ projects, when he would receive between 100–300 shillings a day (US $1.2 to $3.5) Occasionally he found himself without any of these opportunities, and at these times he sold bottled drinks (‘sodas’) from a stand at the entrance to Youth For Life, which was owned by one of its employees. Alongside this rather piecemeal livelihood, he pursued several courses of further training and education, which he paid for himself in small installments. These included a computer course (until the college organizing it went bankrupt and disappeared overnight, taking his money with it), a course in ‘community health’ offered by a college in Kisumu (which he never finished, as he could not continue to pay the fees), and a course in ‘HIV counseling and testing,’ offered by a counseling college. He managed to complete this last course, and it is his narration of the euphoric moment when he eventually gained this publically recognized and highly sought-after certificate, after five years of volunteering and pursuing piecemeal projects, that I have quoted previously. With the certificate, he was able to act upon his contacts within Youth for Life, and he applied for and secured a one-year job as an ‘HIV counselor.’ By this time his wife had given birth to their first child and he was saving up to bring *Ayie* (‘I accept’) gifts of money to her father's home. First, however, he intended to save some money, complete his course in community health, secure a diploma, and help his wife to enroll for a counseling course herself. He was hopeful that, with his new job, he would be able to pay the fees little by little, and that his one-year contract with the NGO would be extended.

When I began my research in Kisumu in 2008, I was struck by the degree to which HIV and biomedical knowledge related to it infused the city. This took shape materially—in newly built HIV clinics, youth centers, and NGO offices running HIV-related projects; in the density of clinical trials and public health research groups operating in and around the city; in posters and billboards advising people to ‘know your HIV status,’ advertising college courses in ‘HIV counseling’; or offering information about projects, jobs, and workshops on HIV-related interventions. It also took shape in the aspirations, identities, lives, and imaginations of many of the city's residents. While HIV is still surrounded by secrecy and stigma, particularly in intimate relationships between women and men, it is also true that knowledge about HIV and the pursuit of this knowledge have become central to life for many of Kisumu's residents, and this in ways beyond those imagined by AIDS policies that encourage people to shape their lives around ‘positive-living’ and proper forms of ‘self-care.’ Such knowledge concerns not only that required for biological survival—the knowledge of one's HIV status, of the virus, how it behaves, how to protect oneself, or how to live with it—but also the ‘exposure,’ as people call it, to particular ways of knowing, speaking, and acting that are associated with particular institutions such as NGOs and their discourses. Here, one's orientation to scientific, biomedical knowledge, and associated discourses concerning healthy and responsible living, marks one out as someone who is ‘moving ahead.’

Due to its association with science, progress, and the future, the pursuit of knowledge about HIV has become the focus of intense desires and forms of self-fashioning among aspiring young people who have secondary school (and sometimes tertiary) education but few prospects of gaining formal employment, let alone a profession. Associating oneself with HIV-related knowledge allows them to gain visibility as actors within HIV interventions. Yet it is not only people like Elias, who try to gain knowledge, recognition, and eventual employment by attaching themselves to sites of intervention. Middle-aged widows and younger men and women who have little formal education and are HIV-positive find that their own ‘exposure’ to HIV, and thus to clinics and clinical trials, opens up opportunities for them to gain knowledge and skills, to create new identities, insert themselves into new social networks, and become attached to new collectives.^2^

In telling me of his struggles over the years to become an HIV counselor with an NGO, Elias returned again and again to two issues. One issue was his pursuit of a certificate and his oft-thwarted attempts to gain one. He had finished secondary school but he had never secured his school-leaving examination certificate, as his father had died the same year, leaving the school fees unpaid, and his relatives had not been able or willing to help him. He had then moved to Nairobi to stay with his older brother, who paid for Elias to begin a computer course, but the tension this caused with his brother's wife forced him to leave. In the following five years, Elias’ struggles to gain a certificate—any kind of certificate, whatever the type or level of qualification—had ended in failure.

The second issue was Elias’ attachment to Youth for Life. Over the years, this attachment gave him something he referred to as ‘exposure’—to new knowledge and new ways of talking and acting, which are associated with both NGOs and the growing prominence of HIV and ‘global health’ interventions in Kenya. According to Elias, his exposure began when, after returning from Nairobi to his parent's rural home, a friend had suggested he come to Kisumu and offered to share his rented room. The friend was a member of the NGO's youth group, and he had got Elias the job of selling sodas from the mobile stand located outside the NGO's offices. By placing himself physically in this particular space, Elias had become visible: he met the people who came in and out of the NGO's offices; he saw the NGO staff (and the volunteers attached to its projects) on a daily basis; and he was able to keep abreast of its activities and to take advantage of any opportunities for workshop attendance and volunteering for projects. Through this exposure, as much as any formal training he received, he learned particular ways of seeing, speaking, acting upon, and also moving through the city and its hinterlands. He could observe and pick up some of the professional language and culture of NGOs (which include many foreign organizations and projects). He also learned to orientate himself to the city as a globalized space of intervention and a ‘truth-spot’ (Gieryn 2006) concerning HIV-related knowledge.

Next, I explore the ‘epidemic of knowledge’ (Boellstorff 2009) about HIV within a city that is dominated by HIV-AIDS and other global health interventions. I am concerned not with the practical, applied question of whether people ‘know’ about HIV and to what extent this knowledge influences their behavior. Nor am I concerned with how the materiality of health knowledge, as displayed in posters and billboards around the city, shapes perception and use (McDonnell 2010). Instead, I examine the value that ‘knowing HIV’ has accrued in a city that has become a focal site of ‘global health’ intervention concerning the virus. I explore the attempts by Elias and other residents to attach themselves to particular spaces and sites associated with globalized knowledge and intervention—such as NGOs running HIV projects, clinical trials, and HIV clinics—to gain ‘exposure’ and access knowledge associated with biomedicine, global health, science, and development. Paying particular attention to the role of certificates, I follow struggles to convert this exposure into a livelihood, an identity, and a future.

In recent years, ‘Global Health’ has become a dominant term for health interventions in the Global South that involve transnational organizations and actors. Drawing transnational resources, expertise, and discourses into particular privileged sites, such interventions inscribe these spaces as ‘global’ while leaving others decidedly ‘local’ (Ferguson 2006; see Sullivan 2011; Wendland 2012). Rolled out in the form of ‘projects,’ interventions also have temporal effects; they offer resources and opportunities, but only for a limited time. Based on ethnographic fieldwork in an East African city that has been inscribed with such spatial and temporal features, this article moves the focus beyond the space of the intervention (the HIV clinic, the hospital, or the project) to consider how these globalized processes shape the hopes, aspirations, and trajectories of Kisumu's residents.^3^

The article was set in motion by the interviews I conducted with around 40 ‘volunteers’ attached to various institutions (mostly NGOs) on various topics concerning their life histories, families and education, their aspirations and motivations, and their experience of volunteering.^4^ Many of these (recorded) interviews read like transcripts of job interviews, as volunteers often initially associated me with the world of NGOs and were eager to make a good impression. As the interviews progressed, however, they also began to voice anxieties and frustrations. The story Elias presented, for instance, was articulate, polished, and emotionally intense. His sentences flowed seamlessly, giving the impression of a practiced performance, and yet, when he narrated how he finally received a certificate, he broke down in tears. Like Elias, I ‘succumbed to the pull of these documents’ (Riles 2006:8), and I have written this article around his and other narratives about knowledge, exposure, certificates, hope, disappointment, and anticipation. I have supplemented this data with observations of and conversations about both projects and life in the ‘HIV city,’ made with volunteers and staff members as well as with patients and other residents of Kisumu, which I conducted over a seven-month fieldwork period.^5^

## GLOBAL HEALTH AND THE ‘HIV CITY’

From the mid-1990s, Kisumu—a city of around half a million in western Kenya—has been produced as a prime site of global health research and intervention. A multitude of organizations, from the transnational to the ‘community-based,’ operate in a variety of partnerships with Kenyan government institutions to target, prevent, treat, and also research HIV/AIDS.^6^ Transnational research organizations (mostly public health institutions from the Global North) run clinical trials of HIV treatment and test interventions such as male circumcision. Since 2004, with funding mainly from the United States, HIV ‘care and treatment’ programs providing free tests and counseling, antiretroviral medicines, and health checks have been made available in HIV clinics (called ‘Patient Support Centres’) in government and NGO sites.^7^ Across the city, public health campaigns proclaim that you must ‘know your status,’ while outreach projects encourage lay people to become involved as ‘volunteers’ in promoting the ‘HIV gospel.’ Together, these interventions and activities connect the city and its populations to transnational flows of recourses, medicines, technologies, and expertise. They also locate it as a ‘truth-spot’ (Gieryn 2006)—a site of knowledge production and its global circulation, in this case, concerning HIV.

In the process, Kisumu has been transformed from a sleepy backwater, mostly bypassed by labor migrants on their way to Mombasa or Nairobi (Geissler & Prince 2010), into a center of disease intervention and research, donor funding and NGO projects, which employs managers, scientists, accountants, and consultants, as well as fieldworkers, secretaries, drivers, clerks, community health workers, and HIV counselors. The mass of projects, funding, employment, and training opportunities registers in the institutional landscape—in the new buildings housing NGO projects within Ministry of Health sites, in the HIV clinics, in the numerous NGOs that sprout up, disappear, or expand their activities, and in the growth of college courses in ‘community based development,’ ‘community health,’ and ‘HIV counseling.’ Public spaces in the city, too, are dominated by HIV—billboards and walls are plastered with health education posters, while ‘mobile VCT’ (Voluntary Testing and Counseling) clinics tour market places and streets.

This AIDS economy registers in the growth and increasing visibility of wealth and mobility, middle class consumption and lifestyles among Kenyan professionals and a growing expatriate population (many of whom are employed by NGOs and research organizations), and it congeals in mega-supermarkets, arcades and Internet cafes, fast food restaurants, and evening bars. Fifteen years ago, the streets of Kisumu were trundled over by old cars, decrepit government land-rovers, and the occasional vehicle of a donor-funded project. Today Kisumu is saturated with 4 × 4 s inscribed with the logos of NGOs, donor organizations, and groups conducting HIV research, while the city streets are congested with the newly bought private cars of its middle-class residents. In 2011, the Kisumu International Airport opened, built by the Chinese. Such mobility, wealth and opportunity are not accessed by the majority of the city's inhabitants, however, who live in the equally fast-growing informal settlements and slums, which lack piped water and sewage, rubbish collection, and electricity.

The transnational flows of resources, medicines, technologies, expertise, and knowledge of ‘global health’ produce privileged enclaves of globalized medicine, fed by external resources and surrounded by a sea of under-resourced health care. However, not all movements are similarly bounded. HIV/AIDS policies encourage laypeople to become involved in interventions, addressing them as ‘responsible subjects’ who can become orientated to the ‘truth’ of HIV and can disseminate such knowledge and related skills to others. This circulation of knowledge relating to HIV, backed by resources, has shaped its value. Knowledge of HIV, and even the virus itself, has become an economic resource for people at all levels of the social strata. It has also become a means of claiming a moral identity and moral value.

## CONVERSIONS OF KNOWLEDGE AND VALUE

The majority of Kisumu's residents survive on the informal economy: selling vegetables, charcoal, or second-hand clothes on the streets, transporting water, or working a *bodaboda* (bicycle taxi). Life is precarious. Unstable incomes mean that many families live from one meal to the next while medical costs and funeral expenses leave them economically devastated or heavily indebted. In this environment, the clinic's injunction to ‘live positively’—to take on the ‘HIV gospel,’ as one informant put it, of embracing one's HIV status and ‘taking control’ of one's health and life by managing one's medical regimen, diet, and sexuality—is incongruous, to say the least. Some people, especially young women who are dependent on relationships with men, may have no wish to reveal their HIV positive identities. Still, many appreciate the care and advice offered by the HIV clinics, and for the poor, the material support offered by NGOS to those who are HIV positive can be a crucial lifeline. Managing one's HIV positive identity does not therefore necessarily imply a whole-scale ‘conversion’ to a new identity, akin to religious conversion (although conversion narratives are readily offered, if you ask for them), but rather a more subtle shift between different registers of revealing and concealing, visibility and secrecy. Being positive can be a source of stigma and rejection, but ‘knowing HIV’ can also be of value.

For the salariat—professional people with a monthly salary—HIV is a less ambiguous resource. The NGOs and other organizations involved in HIV research and interventions provide good salaries, training, and perks such as travel and per-diems (albeit less security than government employment). Those who work for these organizations attend workshops across the country, are exposed to new networks and contacts, and have lifestyles aspired to by many. While few outside the professional classes manage to access such salaried employment and the lifestyles that accompany it, people like Elias can make themselves visible by attaching themselves to NGOs and clinics, seeking ‘exposure’ to workshops and seminars, and thereby gaining knowledge and skills. In this way they gain recognition as people who ‘know’ about HIV and can teach others about it. They may be taken on as ‘volunteers’ for outreach or clinic work, through which they gain further contacts, skills, and some (very basic) remuneration. The ability to ‘know’ HIV opens up not only an (unstable and uncertain) livelihood; it allows one to claim a particular identity and gain visibility and mobility within the city's spaces of intervention. Next I trace the trajectories of some volunteers, focusing on their attempts to position themselves as actors within this economy of knowledge and intervention.

## KNOWING ‘WHO I AM’

Omondi is a community health worker who lives with his wife Star and four children in a solid two-roomed rental house in one of Kisumu's informal settlements. He works at the newly expanded HIV clinic of an NGO, where he registers patients, conducts basic health checks (such as weight and pulse rate), asks them about their health and treatment adherence, and updates patients’ files. In the afternoons, he visits or ‘follows up’ patients who are too ill to come to the clinic or who have not turned up to their appointments.

After an introduction by the director of the NGO, Omondi invited me to accompany him on some of his follow-up visits. The first time we did this, as we threaded our way through one of Kisumu's informal settlements, he told me he was HIV positive. He had discovered his status in 2004, when his wife Star, who was then pregnant, tested positive, and took him to get tested too. His CD4 counts were still relatively high and, he told me, he was “doing well.” I was struck by his desire to talk about his own status, and by the language he used: “I know who I am, the person I am, I know who my wife is and who my child is.” On subsequent occasions, Omondi would often talk about how ‘knowing HIV’ had changed his life and transformed his marriage: “Before, I did not know, we hid from each other. Now we are open with each other.” Moreover, ‘knowing’ himself had opened up a new trajectory. As a young man, he had completed secondary school and started a college course, supported by his parents, until the family had run into hard times. He had been forced to return to his rural home and farm, where he met Star and started a family. Frustrated with rural life and unable to use his education, he had turned to alcohol, and Star left him for another man. She eventually returned to Omondi, and the family moved to Kisumu, where she became pregnant with her fourth child. Life remained hard though. As Star told me,

[At that time], I was just about to cry… we had no job, no money. Omondi would get 20 bob [20 Ksh or US $0.40] a day for carrying a sack of *sekumaweke* [green vegetables], and we would have to eat our daily meal from that money. We could not afford breakfast or any evening meal. We were staying in a house for 400 shillings [US $8 a month] and it was difficult to find the rent. So he left that work and started doing *bodaboda* [bicycle taxi]. [Later] he left that too. Why? He got a stroke, you've seen his eye? One eye is not good. I think it was the cycling, and the stress. You see he is a learned person, he finished form four and went to college and started architecture, and then all his documents got lost, so now he does not even have a secondary school certificate, someone stole his bag and all the documents got lost.

During her pregnancy, Star discovered her HIV status and enrolled in a Prevention of Mother-to-Child Transmission (PMTCT) clinic. She joined an HIV positive ‘support group’ and became a volunteer ‘peer counselor,’ spending one day a week talking to pregnant women at the antenatal clinic. She told Omondi of her status, and after initial resistance, he too took an HIV test. This was a turning point, as he narrated it to me. Encouraged by his wife, he joined the support group that met at the NGO-run health center near their lodgings. He became an ‘active’ member of the group, regularly attending the meetings and becoming one of its leaders. The manager of the NGO's health clinic sent him on a training course in ‘HIV/AIDS counseling,’ which gave Omondi a much-coveted certificate. With the certificate and through the contacts between the NGO, he managed, like his wife, to get an unpaid position as a ‘volunteer’ counselor at the new HIV clinic attached to the Provincial hospital in Kisumu. Omondi spent almost three years as a volunteer, gaining a little remuneration (100 shillings (US $1.80) a day if he got work),^8^ which he supplemented by running a bicycle taxi, while his wife sold charcoal, vegetables, or homemade chapatti. Omondi told me that he much preferred volunteering: “I learned so much from that work. I went for further training.” He also continued to be an “active” member of the support group and the NGO sent him on several workshops, for each of which he gained a certificate of attendance. When the NGO began to expand its HIV clinic in 2007 with funds from the US Presidential Emergency Fund for AIDS Relief (PEFPAR), Omondi, armed with more certificates—one in ‘community health work,’ another in ‘adherence counseling’—gained a position as a community health worker with a monthly salary of 11,000 Kenyan Shillings (approximately US $125) on a one-year contract.^9^

Many people like Omondi and Star attach themselves in similar fashion to clinics, NGO projects, or community-based groups. Few, however, manage to gain employment. Mariam, for example, had been a volunteer for several projects. She was a widow and lived with the orphaned children of her sister and her own youngest child, who was HIV positive. She had found out her own status after being forced by her husband's family to leave their rural home. A friend had taken her to get an HIV test and introduced her to a patient support group attached to the provincial hospital. Like Star, she became a volunteer ‘peer counselor’ at the hospital's HIV clinic, advising pregnant women about HIV at the antenatal clinic. An NGO trained her as a community health worker, and another NGO enlisted her as a ‘community mobilizer’—someone who walks around a neighborhood and talks to people about HIV prevention and treatment. Later, the hospital started a tuberculosis program and used her as a volunteer ‘tracer,’ connecting patients to the clinic and checking on their adherence to medication. Like her attempts to sell vegetables in her own neighborhood, the income she gained from her attachments to these interventions was unpredictable: some projects paid 100 shillings per day, others 3000 per month (US $50), others promised money that never materialized. Still, she insisted that knowing about HIV had enabled her both to live and to make a living: “I just tell people, this is who I am.”

## “WE ARE HIV GRADUATES”: EXPOSURE AND ENLIGHTENMENT

For Mariam, the act of positioning herself within spaces of HIV interventions as a ‘volunteer’ provided a source of income, although it was unstable and had to be combined with other kinds of work. Yet volunteering was about much more than income. Mariam was known among her neighbors as someone who had personal connections at the hospital and the clinic, and at NGOs. Her connections were the outcome of years of volunteering and attendance at patient support meetings, workshops, and training days. They were important to Mariam: through her position in this network she had gained a degree of social standing and recognition, albeit negligible economic advancement. Her volunteering enabled her to make a life in the city, to gain some status and recognition, contacts and networks, all of which fed into a livelihood. Meanwhile, Elias’ ‘exposure’ to NGOs, research, and health interventions held out the possibility—however chimerical—of placing him on a trajectory leading to an office job, further education, economic advancement, and social mobility. It was the access to knowledge and skills associated with the global and immersed in narratives of personal ‘empowerment’ that attracted him. Exposure to spaces of globalized intervention open up, he hoped, a future, one that was tantalizingly visible—he glimpsed it in the lifestyles and mobility of NGO staff, expatriates, and globe-trotting anthropologists. It was almost within his grasp—present and yet absent.

Elias, Omondi, and Mariam often referred to themselves as ‘HIV graduates’ and as being both ‘enlightened’ and ‘empowered.’ This talk of graduation, enlightenment, and empowerment suggests that the process of attaching oneself to a project, navigating the space of intervention, learning new knowledge and skills and gaining certificates, is also a process of fashioning the self as a particular kind of actor, one who moves at ease across the city's global health landscape. The term ‘empowerment’ is closely associated with development and global health interventions (and there is no vernacular translation, people simply insert the English term, *Asebedo empowered*, “I've been empowered”), while the meanings of ‘graduation’ and being ‘enlightened’ refer to the experience of western Kenyans since the early twentieth century with modern education and Christian conversion as a means of self-making and a route to social mobility. Christian converts referred to themselves as ‘walking in the light’ (this is still a common expression), and the contrast between the darkness of the past and the brightness of the present and future resonates with the globalized meta-narratives of ‘positive living’ through knowing the truth of one's HIV status (Prince 2007, 2011). Since colonial times in Kenya, formal education has been intricately linked to modern ideas about health and bodily habits, and, until recently, it has created a pathway to social mobility. HIV is tied to officially sanctioned knowledge mediated by the state and by powerful NGOs, funds, and policies. Knowing HIV is deeply entwined with modern education and modern identities, which, for many people, still hold out the promise of ‘moving ahead’ in life.^10^

Economic empowerment is thus not the sole motivation for people who try to attach themselves as volunteers. They are also attracted by exposure to knowledge itself and to the network of contacts that may open up within and beyond the city, to England and America. The yearning for knowledge is underlined in Omondi's appreciation of his years spent volunteering: “I learned a lot.” Another widowed volunteer, Pamela, joked about her certificates of attendance at HIV workshops, “I have graduated, and people think I'm going to America!”

The knowledge one gains from attending workshops and training days is, however, very different from that associated with formal education leading to a college diploma, a degree, or professional training. It is more ephemeral. Moreover, it appears more important in its form than its content. Next I take a closer look at the role of certificates in this economy of knowledge.

## CERTIFICATES

Unlike Omondi, Mariam had no secondary school certificate but she had gained several certificates of attendance at various HIV-related workshops and seminars. Every inch of wall space in her single rented room, divided into bedroom and living room by a hanging curtain of material, was covered with posters about HIV and AIDS, proclaiming messages such as ‘Know your status’ and ‘Know your rights.’ A large poster displayed two photographs of the same person, ‘before’ and ‘after’ antiretroviral treatment; another showed Barack Obama getting an HIV test when he visited Kisumu as a US senator in 2006. Several framed certificates held pride of place next to a photograph of Mariam at a training workshop on ‘peer counseling’ about HIV. She showed me photocopies of these and other certificates, carefully wrapped up in a plastic bag and locked up in a small cabinet next to her bed. These documents certified her attendance at workshops and training programs; each bore the insignia of the NGO, government ministry, and donor agency involved.

Mariam's hoard of certificates was not unusual. People often showed me certificates, usually wrapped up in a plastic bag and kept in a safe place in the house. As Elias’ narrative reveals, certificates are repositories of much hope and anticipation, as well as pride. Not everyone hoards these certificates, of course, but many people working in the informal or *juakali* economy, defined by physical labor ‘under the hot sun,’ aspired to less exhausting ‘office work.’ They talked to me about their lack of opportunity in terms of missing a vital certificate, such as the school-leaving certificate, and their desire to gain some ‘training’ and ‘move ahead.’ Even those with monthly salaries and office jobs, whom Kenyans refer to as the ‘working classes,’ are not immune to this desire for further knowledge and skills. Many are enrolled in college courses and are pursuing diplomas or further degrees. New colleges have been established and existing colleges have expanded to offer a range of courses—in computing and IT, community development, pharmacy, and HIV counseling—from certificates to diplomas to degrees. Many employees enroll in several different courses consecutively or concurrently, and colleges offer flexible timetables, such as night classes, vacation studies, and long-distance learning courses, to accommodate people's working hours. Staff members of NGOs and research organizations often stay in the office after hours to use the computer for study purposes. Although they often enjoy good perks and higher salaries than government employees, they are mostly on one-year contracts, with little economic stability, and their future is uncertain. Building up a profile of courses and qualifications, they hope, will make them more attractive to their employers.

For those, like Elias, Omondi, or Miriam, who lack professional qualifications and formal employment, and who cannot afford diplomas or degrees, certificates that give evidence of exposure to spaces of global health intervention are highly valued documents. Such certificates are part of a broader economy of development (Pigg 1997) which focuses on ‘grassroots participation’ or ‘capacity building’ through the development of ‘human capital’ (see Pfeiffer 2003; Li 2005). Workshops, seminars, and other training courses distribute certificates to all participants (see Pfeiffer 2003), often with elaborate graduation ceremonies at the end of these activities. Even clinical trial subjects expect to be given certificates that attest their participation and recognize their exposure to a particular kind of knowledge. Advertisements for positions such as ‘data clerk’ or ‘HIV counselor’ demand evidence of certificates, and applicants submit bundles of them. There are rumors about the production of fake certificates and attempts to buy and sell certificates.

Certificates are the focus of much care, hope, pride, and anticipation because they make a claim to knowledge. But on closer inspection, this claim appears rather empty. Workshops on ‘HIV prevention’ or ‘treatment’ provide little information that laypeople do not already know: HIV is a virus transmitted through sexual contact or through the blood, and HIV can be transformed into a life condition through careful self-management of diet, sexuality, and pharmaceutical regimes. What people do learn is the ability to speak about and transmit this knowledge through a particular—global—language, and in doing so, to project a particular identity. Certificates give evidence of this ability and mark the holder as someone who has been ‘exposed.’ They lay claim to a kind of ‘technical know-how’—underlined in the language of ‘graduation’—but also to ‘technical know-who’ (Whyte 2002), as they mark one's personal connections and access to institutions. Certificates thus provide evidence of a person's navigation of spaces of intervention, in which globally circulating knowledge is channeled into particular sites. They extend a person's capacity to be seen, heard, and recognized, and to move within and across these spaces. Certificates are thus intimately involved in processes of self-fashioning and identity making. Yet because the skills and qualifications encapsulated in these certificates are ephemeral, such processes are fragile and the identity cannot be held onto for very long.

## FRAGMENTATION, TRANSIENCE, AND INSTABILITY

For those outside the professional ‘working’ class, but who are trying to enter it and thereby, as they put it, ‘move ahead’ in life, certificates offer a hope of recognition, which may open up opportunities within the city's globalized interventions. Yet the kind of knowledge that is circulated through training sessions and workshops is quite different from professional knowledge. Each certificate gives evidence of a specific skill: ‘peer counseling,’ for example, or ‘family life training,’ ‘HIV counseling,’ and ‘treatment adherence’ counseling. Knowledge is fragmented, organized as ‘information’ in discrete units, clustered around specific problems and solutions, and tailored to a specific issue (see Bauman 1992). The value of this knowledge is unstable and uncertain. Certificates are not durable passports to employment and status. Almost soon as one has gained a certificate in a particular skill, it is made obsolete by new up-to-date knowledge.

As more people acquire these certificates, their value decreases. In Kisumu, ‘HIV counseling’ certificates had a high value when few people possessed them, but their value decreased as people rushed to take courses in counseling. The struggle is then to get a diploma or a degree. In this field, gaining a position, even as a volunteer, is intensely competitive. As the director of a national NGO told me, “There are many who just come by our facility here asking for the same position, to work as volunteers. But they fail to get taken on.” Such organizations may receive 100–200 applications for a position such as data clerk or HIV counselor.

In light of this constantly changing landscape of opportunity, people who have decided to live as openly ‘positive,’ such as Omondi and Miriam, as well as aspiring young people like Elias, must continually position themselves in relation to the circulation of new knowledge, and to do so, they have to maneuver themselves into particular spaces, from which they are visible and available to those in authority. Omondi and Miriam positioned themselves within HIV clinics as ‘good’ clients and ‘active’ members of patient self-help groups, as people able to ‘talk the right talk and walk the right walk.’ This involved cultivating relationships, particularly with those who are in responsible positions, who manage and distribute resources. As already noted, Elias sold sodas outside the NGO, offered himself as a clinical trial subject to a transnational research group, joined a youth group, gained some training in ‘life skills’ relating to HIV infection, began several training courses, and, as he put it, ‘tarmacked’ between various institutions, as he built up a network of contacts and a repertoire of skills, while pursuing various courses and certificates. Along the way, to position himself in the right place at the right time, he had to negotiate a changing landscape of NGOs, research groups, and donor-funded projects.

Elias’ story reveals the importance of navigation, involving physical presence, availability, and ‘exposure,’ through which people are able to convert knowledge explicitly associated with global health interventions. But his story also points to the instability and uncertainty of these trajectories, as projects fail, resources run out, and colleges disappear. In a landscape of tenuous projects, unstable flows of donor funds, unreliable training programs, and intense competition, projects can be closed down and jobs lost in the space of a few months, and people must return to making do by combining multiple forms of income—selling sodas or second-hand clothes, making chapatti or offering bicycle taxis. The institutional partnerships that produce Kisumu's global health interventions are themselves unstable, dependent on the flows of external funds and on relationships between donors, the Kenyan government, research groups, and various NGOs. Colleges, projects, and interventions may disappear overnight. The one breakthrough that Elias experienced during his five years of ‘tarmacking’ between the youth group, the clinical trial, and the soda stand was when he was taken on as a volunteer ‘district coordinator’ for schools within a project called ‘Straight Talk’: for this he received a regular income of 4000 shillings a month. The project was funded by the German development agency and organized through a national NGO. But when this project came to an abrupt standstill, amidst accusations of management mishandling funds, Elias lost work literally overnight. In recounting the episode to me, he said with a sigh, “So I lost everything, and I could not continue that college course I was following. God knows. I then had to look for an alternative.” He returned to his soda stall and it was some months before his contact with a teacher in a local college gave him a lucky break: the opportunity to pursue an HIV counseling course, and to pay for it in small installments.

## CONCLUDING THOUGHTS: VISIBILITY, RECOGNITION, AND ANTICIPATION

In Kisumu, as across much of eastern and southern Africa, global health interventions, rolled out in response to the HIV/AIDS epidemic, involve intense efforts to persuade individuals to shape their lives around new regimes of biomedical knowledge. A growing body of scholarship examines how such biomedical regimes and technologies bring into play new rationalities and temporalities, new subjectivities and ‘biosocial’ collectives orientated around forms of self-care (Biehl 2007; Nguyen 2005, 2010; Petryna 2002; Rose 2007). Ethnographic research shifts attention away from the Foucauldian transformations implied in these processes to explore how people put biomedical technologies to work not only, or necessarily, upon their selves, but also, or primarily, upon their social worlds: to create identities, demand recognition, and make claims on both intimate relations and social collectives (Marsland 2012; Meinert, Mogensen, and Twebaze 2009; Marsland and Prince 2012; Prince 2012; Street 2012).

In this article, I have examined the movement, diversification, and conversion of biomedical knowledge beyond forms of self-care, as it enters people's lives in intimate ways. My material suggests that knowledge of HIV is not necessarily inward-looking, used as a technology of the self to shape biological survival and create a ‘responsibilized subject.’ It is also outward-focused, shaping the knower in a wider field of recognition, as an actor within globalized interventions and an ‘enlightened’ person. Here, one's ‘exposure’ to biomedical knowledge and associated spaces and projects of intervention is a form of ‘empowerment.’ It enables the actor to place herself in a forward-moving trajectory. This process is captured in two expressions I often heard among volunteers, project staff, and other city residents: “I am developing myself,” and “I am moving ahead” (Prince 2013). While these orientations to spaces of knowledge and intervention do not empower people to take control of their health and lives in the way that policymakers imagine, they enable people to gain visibility, connections, recognition, and respect.

Certificates operate as extensions of such capacities. They are highly affective documents, deeply entwined with identities and relationships and invested with hope and anticipation (see Gordillo 2006; Navaro-Yashin 2007; Riles 2006; Street 2012; Yoltar 2009). They can project a person's identity as a virtuous subject of development. They extend and materialize connections, provide an interface between individuals and more powerful actors and institutions, and anticipate a future trajectory. They gain value not only from being displayed on a living-room wall but also from being circulated in the NGO economy, where they make the holder visible, project her capacities and connections, demand recognition, and, hopefully, elicit a response. Yet certificates are also inherently fragile documents. They are difficult to get hold of and even when grasped, their value easily deprecates. Although they enable their holders to project a form of agency, it is limited and circumscribed. Tied to a shifting landscape of intervention, the value of certificates is uncertain and precarious. They may elicit only a fleeting recognition and can be reduced at any time to inert pieces of paper.

Why do people invest their identities, hopes, and futures into gaining knowledge and accumulating certificates when their value is so uncertain? Certificates provide evidence of their holder's having embraced a key message of HIV intervention: the empowerment of the self through becoming responsible for one's health, life, and future. They mark an individual's status as a ‘proper’ subject of development (Pandian 2008). In a shifting terrain, where any opportunity for advancement appears transient and ephemeral, perhaps the only option available is to invest into oneself and one's ‘human capital,’ as defined by the global health economy (Li 2007). The search after and accumulation of knowledge through certificates is one of the few options available for building up a portfolio of skills, investing in one's capacities, and demonstrating one's flexibility—the ability to respond to new opportunity, to navigate a constantly changing terrain (see Feher 2009). That the terrain on which one build's one's human capital is constantly changing only intensifies this desire to access knowledge and gain certificates.

The production of Kisumu as a truth-spot of knowledge about HIV thus feeds into a particular ‘politics of temporality and affect’ (Adams, Murphy, and Clarke 2009:246). This is defined by a state of anticipation, both embodied and engendered by certificates. While anxiety, uncertainty, frustration, and disappointment are features of this state, Elias, Marian, and Omondi all display a hopeful anticipation toward converting an orientation to biomedical knowledge and attachment to globalized spaces of intervention into a future, within the city and beyond it.

## NOTES

1. Pseudonyms are used.

2. From its beginnings, questions of knowledge have been central to the HIV-AIDS epidemic, both to understanding it and to responses to it, at all scales from the individual to scientific research and policymaking (see Boellstorff 2009).

3. Cities have always been important sites for the formation of identities, from late colonial and independence concerns with shaping of modern, metropolitan subjects (e.g., Slaughter 2004) to the postcolonial disintegration of both urban infrastructure and linear narratives of progress and their association with the “crisis” or “abjection” of the urban African subject (Ferguson 1999; Mbembe and Roitman 1995). Some scholars analyze the city as a “suffering body” (Le Marcis 2004) and as a “wounded” place (Till 2012), littered with displacements, exclusions but also possibilities for repair and healing.

4. Following wider trends in development practice, the Kenyan government and foreign donors have promoted the use of volunteers in HIV interventions since the late 1990s. Figures on volunteers are hard to come by, perhaps because they occupy a grey area between formal and informal work. However, a national survey conducted by the NGO coordination board of registered NGOs cites a figure of 90,411 volunteers who are attached to the 1334 NGOs that responded to the survey (compare this to the figure of 14,217 official staff members) (http://www.ngobureau.or.ke/Publications/National%20Survey%20of%20NGOs%20Report.pdf). Most volunteers have no work contract, and while they expect some degree of remuneration, there is no standard to this and thus considerable exploitation.

5. The article is based on fieldwork on HIV and related interventions in Kisumu, which took place from October 2008 to April 2009, with additional shorter visits in April 2008, December 2009, and September 2010. Apart from interview material, I draw upon longer-term acquaintances with some volunteers, whom I accompanied to family events and church meetings as well as to the community-based, patient-support, youth and women's groups of which they were members. I also interviewed and followed staff and patients associated with an NGO-run HIV clinic located on the edge of one of Kisumu's informal settlements, and with an HIV clinic within an urban government hospital, and made regular visits to other NGO-run projects, government clinics, and community-based groups in the city and its hinterlands.

6. The last count, in 2008, gives a figure of 907 non-governmental or ‘community-based’ organizations operating in the city and its hinterlands. See the 2007–2008 report by the NGOs Coordination Board: http://www.ngobureau.or.ke/Publications/National%20Survey%20of%20NGOs%20Report.pdf. It shows that there are 117 NGOs with their headquarters in Kisumu, and 790 operating in Kisumu but with their headquarters elsewhere. See also http://www.kanco.org/KANCOmembers.php

7. These clinics operate within both NGOs and Ministry of Health sites; and the procurement and distribution of medicine, staff salaries, provision of training, and material support are organized through by partnerships between the government, non-governmental and transnational organizations.

8. At the time of this conversation, in 2009, the price of a 2 kg bag of ugali flour, the staple food in western Kenya, had risen to 120 Kenyan shillings (US $1.5).

9. Adherence counseling involves educating people about treatment regimens and management of one's life on antiretroviral drugs.

10. Not everyone embraces this desire to position oneself as a visible actor in relation to HIV knowledge and biomedical science. I met some and heard of others who rejected an HIV diagnosis and the technologies of tests and medicines (often they were the male partners and husbands of the women we met at the HIV clinics and patient support groups). However, such is the dominance of biomedical knowledge that those rejecting it have to position themselves in relation to it (Callon and Rabeharisoa 2004; Steinberg 2008).
